# International metastatic renal cell carcinoma database consortium classification and regression tree analysis to characterize objective response rates to first line in metastatic renal cell carcinoma

**DOI:** 10.3389/fonc.2026.1827686

**Published:** 2026-05-04

**Authors:** Martin Zarba, Dylan O’Sullivan, David Maj, Winson Y. Cheung, Lisa Ludwig, Connor Wells, Evan Ferrier, Razane El Hajj Chehade, Frede Donskov, Marc Eid, Sumanta Kumar Pal, Benoit Beuselinck, Rana R. McKay, Lori Wood, Jae Lyun Lee, Cristina Suárez, Kosuke Takemura, Ignacio Duran, Toni K. Choueiri, Daniel YC Heng

**Affiliations:** 1 Department of Medical Oncology, Arthur J.E. Child Comprehensive Cancer Centre, Calgary, AB, Canada; 2 Cumming School of Medicine, Department of Oncology, University of Calgary, Calgary, AB, Canada; 3 Department of Medical Oncology, IPSEN, Edmonton, AB, Canada; 4 Dana-Farber Cancer Institute, Harvard Medical School, Boston, MA, United States; 5 Department of Medical Oncology, University Hospital of Southern Denmark, Esbjerg, Denmark; 6 Department of Medical Oncology, City of Hope Comprehensive Cancer Center, Duarte, Canada; 7 Department of Medical Oncology, UZ Leuven, Leuven, Belgium; 8 Department of Medical Oncology, UC San Diego School of Medicine, San Diego, CA, United States; 9 Queen Elizabeth II Health Sciences Centre, Dalhousie University, Halifax, NS, Canada; 10 Asan Medical Center, University of Ulsan College of Medicine, Seoul, Republic of Korea; 11 Medical Oncology, Vall d’Hebron Institute of Oncology, Vall d’Hebron University Hospital, Barcelona, Spain; 12 Department of Genitourinary Oncology, Cancer Institute Hospital, Japanese Foundation for Cancer Research, Tokyo, Japan; 13 Hospital Universitario Marqués de Valdecilla, IDIVAL Santander, Santander, Spain

**Keywords:** classification and regression (decision) tree analysis, IMDC, immunotherapy combined therapy, machine learning, renal cell carcinoma

## Abstract

**Introduction:**

Therapies for metastatic renal cell carcinoma (mRCC) have evolved significantly, making treatment decisions more complex. We used machine learning (ML) to identify subgroups of patients who have a high probability of response to first line systemic treatment.

**Methods:**

Patients from the International mRCC Database Consortium (IMDC) with mRCC and treatment response measured to first-line were identified, and a ML classification and regression tree analysis was conducted, in which we grew a complex tree up to a depth of 30 with a minimum node split size of 2 with no constraints on the cost-complexity parameter. The resulting tree was pruned according to the cost-complexity parameter that minimized the leave one out cross-validated error rate and had a minimum bucket size of 25 patients.

**Results:**

2,549 patients were included, 73.2% male, 13.5% non-clear cell histology, 70.3% nephrectomy. 19.4%, 54.2%, and 26.4% had favorable, intermediate and poor IMDC risk respectively. First line treatment regimens consisted of VEGF inhibitors (51.5%), IO-IO combinations (32.3%), and IO-TKI combinations (16.2%). The ORR was 36.0% overall, with 29.6% for VEGF inhibitors, 39.1% for IO-IO, and 50.2% for IO-TKI combinations. ML identified 5 hierarchal variables, therapy type, nephrectomy, lung metastasis, other sites of metastasis, and age, that divided patients into 7 different categories with different response probabilities. VEGF therapy showed the poorest response, with no additional variables able to predict response. The highest ORR was observed in patients treated with IO-TKI and nephrectomy (54.9%); and in those treated with IO-IO, nephrectomy, and only lung metastasis (59.8%). Factors associated with poorer ORR included non-clear cell histology, older age, bone and liver metastases, poor performance status, elevated neutrophils, and poor IMDC risk score.

**Conclusion:**

This large-scale ML analysis identified five key clinical variables that predict treatment response in mRCC, with treatment type emerging as the primary determinant. These results suggest that treatment selection for mRCC could potentially be optimized by considering these hierarchical variables, though further validation is needed.

## Introduction

Treatment strategies for metastatic renal cell carcinoma (mRCC) have seen a significant change in the past decade mainly because of the first-line use of immunotherapy combinations (1, 2). These combinations have emerged as the new standard of care for these patients in the first line setting, either as dual immunotherapy with nivolumab and ipilimumab (IO-IO) or with immunotherapy plus a vascular endothelial growth factor receptor tyrosine kinase inhibitor (IO-TKI), as they have showed significant improvement in outcomes compared to the previous standard of care, sunitinib. Pivotal randomized trials using IO-IO (3) and IO-TKI combinations (4–7) have consistently demonstrated clinical success in this setting. These advances have transformed the therapeutic landscape of mRCC, offering durable responses and long-term survival for a subset of patients (8).

Despite these improvements, selecting the optimal first-line therapy remains a major challenge (2). The IMDC prognostic model, developed in the vascular endothelial growth factor receptor therapy era (VEGF), has long been the cornerstone for risk stratification and remains prognostic with modern immunotherapy regimens (9). However, it was not designed to predict response to specific treatment modalities. In clinical practice, physicians continue to rely on empirical decision-making (10), favoring IO-TKI combinations for symptomatic patients requiring rapid tumor shrinkage and IO-IO regimens for those asymptomatic patients with sarcomatoid features or intermediate/poor-risk disease, based on assumptions drawn from cross-trial comparisons and secondary endpoints (11). This approach, while pragmatic, lacks precision and does not fully account for the biological heterogeneity of mRCC (12, 13).

To refine treatment selection, there is a pressing need for more sophisticated tools capable of integrating clinical and biological data to uncover patterns of response beyond traditional prognostic scores (14). Machine learning (ML) offers an opportunity to move beyond predefined risk groups, allowing the identification of previously unrecognized patient subpopulations that derive the greatest benefit from specific therapeutic approaches (15–19).

The International Metastatic RCC Database Consortium (IMDC) provides a robust platform for such analyses, as it prospectively collects harmonized clinical and laboratory data from academic and community centers across North and South America, Europe, Asia, and Australia. Leveraging this large, real-world dataset, we applied ML algorithms to identify subgroups of patients with distinct responses to different treatments, aiming to better understand the determinants of therapeutic benefit in the contemporary mRCC landscape.

## Methods

### Study design and population

A retrospective analysis was conducted using the IMDC to identify patients with mRCC, irrespective of histology, who initiated first-line systemic therapy between January 2015 and December 2024. Data were collected from hospital and pharmacy records using standardized database collection templates. Institutional review board approval was obtained from each participating center.

This study included patients with mRCC that were treated either with VEGF, IO-IO or IO-TKI in first line between 2015 and 2024 and had treatment response measured. Only contemporary, guideline-recommended treatment regimens reflective of real-world clinical practice were included. IO-TKI combinations were defined as follows: axitinib plus pembrolizumab, cabozantinib plus nivolumab, lenvatinib plus pembrolizumab, or axitinib plus avelumab. These combinations are based on the approved IO-TKI combos on the different jurisdictions that are part of the IMDC. VEGF was defined as sunitinib, pazopanib, or cabozantinib. IO-IO was defined as ipilimumab plus nivolumab. Patients were excluded if they received any first-line treatment outside these regimens or if IMDC risk classification could not be determined due to missing data.

### Outcomes

The primary endpoint of this study was the identification of predictors of response (partial or complete response by investigator assessment), identifying variables that divide patients into different categories with different response probabilities. Secondary endpoints included overall response rate (ORR), treatment duration (TD), time to next treatment (TTNT), and 18-month overall survival (OS) in the different identified subgroups of patients. The 18-month OS landmark was selected based on its use as a clinically meaningful landmark in pivotal phase III trials of first-line IO-based combination therapies in metastatic RCC, where 18-month OS rates were consistently reported.

Associations between baseline patient, disease, and treatment characteristics and treatment response were evaluated using univariable logistic regression models. For each covariate, a separate logistic regression model was fitted with treatment response (defined as complete or partial response) as the dependent variable. Results were reported as odds ratios (ORs) with corresponding 95% confidence intervals (CIs). Analyses were performed in the overall study population and in prespecified subgroups according to treatment category (VEGF-based therapy, IO–IO, and IO–TKI). Patients with missing data for a given variable were excluded from the corresponding univariable analysis.

To identify subgroups of patients who have a high probability of treatment response, we conducted a ML Classification and Regression Tree (CART) analysis. For the CART analysis we grew a complex tree up to a depth of 30 with a minimum node split size of 2 with no constraints on the cost-complexity parameter. The resulting tree was pruned according to the cost-complexity parameter that minimized the leave one out cross-validated error rate and had a minimum bucket size of 25 patients (>1% of the sample).

OS was defined as the time from initiation of first-line systemic therapy to death from any cause, with censoring at the last follow-up. TTNT was defined as the time from first-line therapy initiation to the initiation of second-line therapy or death, with censoring at the last follow-up. TD was defined as the duration of first-line systemic therapy until discontinuation for any reason, with censoring at the last follow-up. TTNT and TD were chosen over progression-free survival (PFS) or time to treatment failure (TTF) due to the potential for sustained immunotherapy effects beyond treatment discontinuation and the possibility of treatment beyond first progression.

Response to treatment was determined based on physician assessment using Response Evaluation Criteria in Solid Tumors (RECIST) v1.1 principles. ORR was calculated as the proportion of patients achieving either complete response (CR) or partial response (PR) as their best response to first-line systemic therapy, according to local investigators.

For all the analysis, including the CART analysis, a complete-case approach was used; patients with missing values in any variable entered into the model were excluded.

### Statistical analysis

Comparisons of patient outcomes were performed within each subgroup. OS, TTNT, and TD were estimated using the Kaplan-Meier method, with significance assessed by log-rank test (p < 0.05). Baseline demographic and clinical characteristics were summarized using proportions for categorical variables and medians with interquartile ranges for continuous variables. Chi-square tests were used to assess differences in baseline characteristics between groups, with statistical significance set at p < 0.05. All statistical analyses were performed using SAS statistical software (version 9.4; SAS Institute Inc., Cary, NC, USA).

## Results

### Population and patient characteristics

A total of 2,549 patients were included with a mean age of 63.9 years (SD 10.9 years). Of these, 73.2% were male, 13.5% had non-clear cell histology, 12.2% had sarcomatoid differentiation, 70.3% had a nephrectomy, and 19.4%, 54.2%, and 26.4% had favorable, intermediate and poor IMDC risk respectively. First line treatment regimens consisted of VEGFR inhibitors (VEGF) in 51.5% of the population, IO-IO combinations in 32.3%, and IO-TKI combinations in 16.2%. The baselines characteristics are summarized in **Table 1**. Of patients who had undergone nephrectomy (n = 2,549), 2,270 (89.1%) had the procedure performed prior to the diagnosis of metastatic disease, suggesting the majority represent nephrectomy for localized disease with subsequent metastatic progression rather than cytoreductive nephrectomy.

**Table 1:** Baseline characteristics.

Variables	Overall
	n = 2549 (%)
Mean Age at treatment initiation (SD)	63.9 (10.9)
Male	1867 (73.2)
Non clear cell histology	343 (13.5)
Sarcomatoid differentiation	312 (12.2)
Nephrectomy	1792 (70.3)
Multiple Metastasis	1903 (74.7)
Kidney Metastasis	417 (16.4)
Lymph Node Metastasis	1195 (46.9)
Lung Metastasis	1770 (69.4)
Brain Metastasis	185 (7.3)
Liver Metastasis	458 (18.0)
Bone Metastasis	849 (33.3)
Adrenal Metastasis	359 (14.1)
Pancreas Metastasis	182 (7.1)
Pleural Metastasis	131 (5.1)
IMDC risk group	
Favorable	494 (19.4)
Intermediate	1382 (54.2)
Poor	673 (26.4)
Karnofsky Performance Status < 80%	437 (17.1)
Less than 1 year from diagnosis to systemic therapy	1635 (64.1)
Hemoglobin < lower limit of normal	1249 (49.0)
Neutrophils > upper limit of normal	364 (14.3)
Platelets > upper limit of normal	419 (16.4)
Corrected calcium > upper limit of normal	304 (11.9)

SD, standard deviation; IMDC, International metastatic renal cell carcinoma database consortium.

The ORR for the overall population was 36.0%. Amongst subgroups, the ORR was 29.6% for VEGF inhibitors, 39.1% for IO-IO, and 50.2% for IO-TKI combinations. The predictors of treatment response (complete or partial) among patients with mRCC treated with first-line VEGF, IO-IO, or IO-TKI are summarized in **Table 2**.

**Table 2:** Predictors of treatment response (complete or partial) among metastatic RCC patients treated with first-line VEGF, IO-IO, or IO-TKI.

Variables	Treatment response (all patients) N = 2549	Treatment response (VEGF) n = 1313	Treatment response (IO-IO) n = 824	Treatment response (IO-TKI) n = 412
	Odds ratio (95% CI)	Odds ratio (95% CI)	Odds ratio (95% CI)	Odds ratio (95% CI)
Treatment group				
IO-TKI	1.00	NA	NA	NA
VEGF	**0.42 (0.33-0.53)**	NA	NA	NA
IO-IO	**0.65 (0.51-0.84)**	NA	NA	NA
Age				
<60	1.00	1.00	1.00	1.00
60-69	0.92 (0.75-1.12)	0.87 (0.64-1.17)	0.94 (0.67-1.32)	0.97 (0.58-1.61)
70+	**0.80 (0.65-0.99)**	0.80 (0.59-1.09)	0.86 (0.58-1.26)	0.62 (0.36-1.05)
Sex				
Female	1.00	1.00	1.00	1.00
Male	1.18 (0.97-1.44)	1.30 (0.97-1.74)	1.08 (0.76-1.53)	1.13 (0.71-1.80)
Non clear cell histology				
No	1.00	1.00	1.00	1.00
Yes	**0.58 (0.44-0.76)**	**0.57 (0.38-0.84)**	**0.60 (0.37-0.97)**	**0.42 (0.22-0.79)**
Sarcomatoid differentiation				
No	1.00	1.00	1.00	1.00
Yes	0.98 (0.75-1.28)	0.74 (0.48-1.12)	1.54 (0.98-2.42)	0.63 (0.32-1.23)
Nephrectomy				
No	1.00	1.00	1.00	1.00
Yes	**1.29 (1.04-1.61)**	1.03 (0.74-1.45)	1.31 (0.91-1.88)	**2.56 (1.41-4.72)**
Multiple mets				
No	1.00	1.00	1.00	1.00
Yes	1.08 (0.86-1.36)	1.09 (0.78-1.52)	0.91 (0.61-1.37)	1.35 (0.75-2.43)
Lung met				
No	1.00	1.00	1.00	1.00
Yes	**1.22 (1.00-1.50)**	1.05 (0.79-1.41)	**1.77 (1.23-2.56)**	0.99 (0.62-1.59)
Liver met				
No	1.00	1.00	1.00	1.00
Yes	**0.73 (0.58-0.92)**	0.74 (0.52-1.04)	0.81 (0.54-1.20)	0.61 (0.34-1.09)
Bone met				
No	1.00	1.00	1.00	1.00
Yes	**0.69 (0.56-0.83)**	**0.67 (0.50-0.89)**	0.81 (0.58-1.14)	**0.62 (0.38-1.00)**
Brain met				
No	1.00	1.00	1.00	1.00
Yes	0.96 (0.68-1.33)	0.96 (0.59-1.53)	0.70 (0.38-1.25)	2.09 (0.78-6.11)
Lymph node mets				
No	1.00	1.00	1.00	1.00
Yes	1.01 (0.84-1.22)	0.95 (0.73-1.25)	1.07 (0.77-1.49)	1.12 (0.70-1.79)
Karnofsky performance status < 80%				
No	1.00	1.00	1.00	1.00
Yes	**0.72 (0.56-0.92)**	0.72 (0.50-1.03)	0.75 (0.48-1.15)	0.60 (0.31-1.14)
Less than 1 year from diagnosis to systemic therapy				
No	1.00	1.00	1.00	1.00
Yes	1.04 (0.84-1.28)	0.83 (0.62-1.10)	1.17 (0.79-1.74)	**1.97 (1.17-3.39)**
Hemoglobin < lower limit of normal				
No	1.00	1.00	1.00	1.00
Yes	0.86 (0.71-1.04)	0.80 (0.61-1.05)	0.98 (0.71-1.35)	1.00 (0.60-1.66)
Neutrophils > upper limit of normal				
No	1.00	1.00	1.00	1.00
Yes	**0.67 (0.50-0.88)**	**0.64 (0.43-0.95)**	**0.56 (0.34-0.89)**	0.97 (0.46-2.04)
Platelets > upper limit of normal				
No	1.00	1.00	1.00	1.00
Yes	1.12 (0.87-1.45)	0.84 (0.56-1.24)	**1.54 (1.03-2.31)**	1.21 (0.56-2.63)
Corrected calcium > upper limit of normal				
No	1.00	1.00	1.00	1.00
Yes	1.10 (0.83-1.44)	1.06 (0.70-1.59)	1.10 (0.70-1.73)	1.10 (0.56-2.19)
IMDC risk score				
Favourable	1.00	1.00	1.00	1.00
Intermediate	0.96 (0.76-1.21)	0.76 (0.55-1.04)	1.29 (0.78-2.16)	1.60 (0.97-2.65)
Poor	**0.75 (0.57-1.00)**	**0.49 (0.33-0.73)**	1.16 (0.66-2.07)	1.50 (0.77-2.97)

IMDC, International metastatic renal cell carcinoma database consortium; met, metastasis. Bold values are used when the Odds ratio and 95% CI do not cross 1, highligting variables that showed a statistically significant difference in odds.

### Primary endpoint

Overall, IO-TKI was associated with better responses compared to VEGF and IO-IO. In the overall population, non-clear cell histology, older age, metastasis in bone and liver, poor performance status, high neutrophil count, and poor IMDC risk score were associated with inferior response rates, while lung metastasis and nephrectomy were associated with better response rates. For the VEGF subgroup, non-clear cell histology, bone metastasis, high neutrophil count and worse IMDC risk score were predictive of poor response. For the IO-IO subgroup, non-clear cell and high neutrophils were predictive of poor response, while the presence of lung metastasis and a high platelet count were predictive of treatment response. For the IO-TKI subgroup, non-clear cell histology and bone metastasis were predictive of poor response; while nephrectomy and less than a year from diagnosis to treatment were predictive of improved ORR.

**Figure 1:**
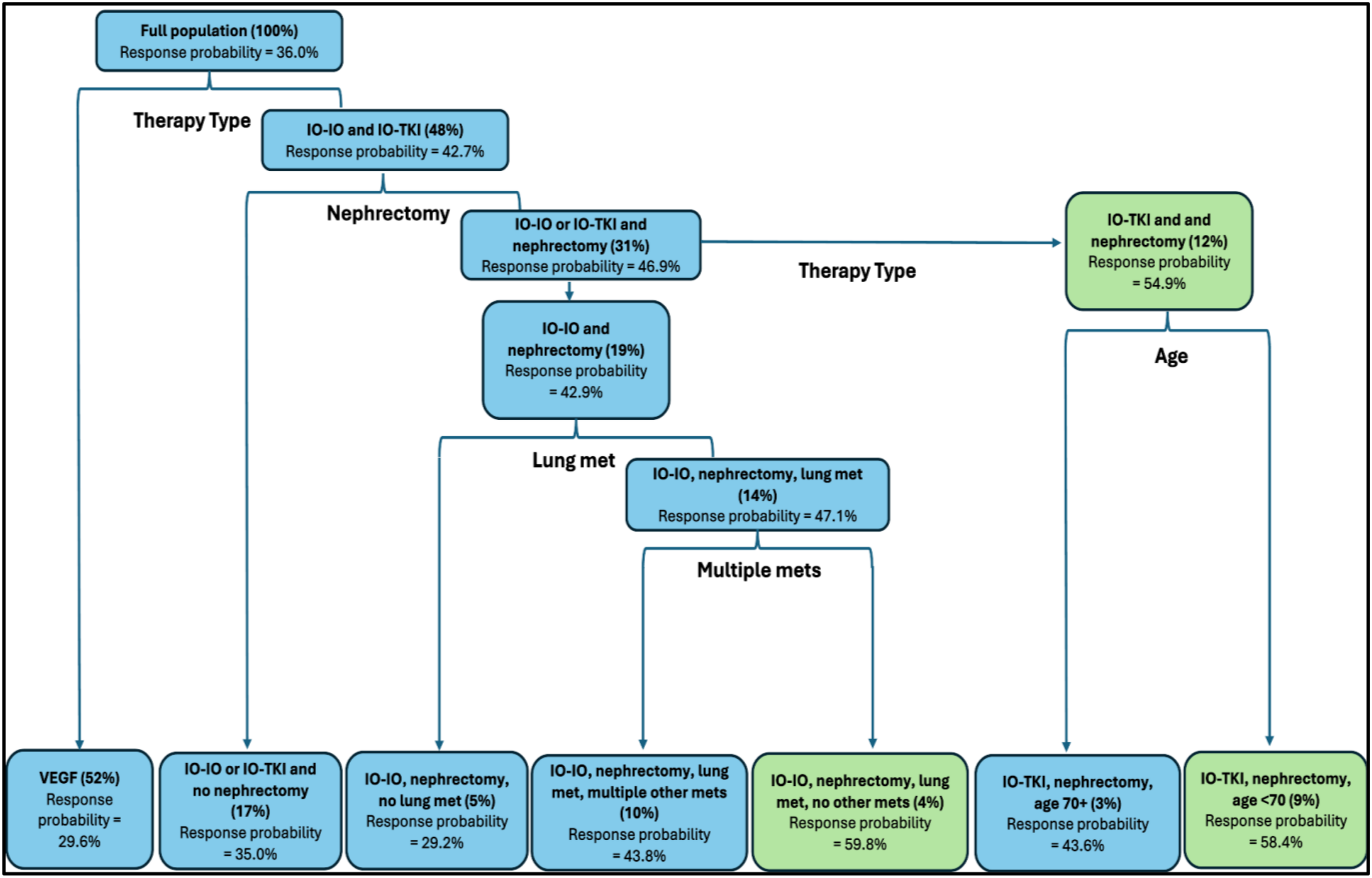
CART analysis. AUC = 0.61 in training dataset and 0.58 in test data. Green boxes represent subgroups with a response probability higher than 50%.

The classification and regression tree analysis done with ML identified 5 hierarchal variables (therapy type, nephrectomy, lung metastasis, presence of other metastasis sites, and age), that divided patients into different categories with different response probabilities (see **Figure 1**). The first division was based on therapy type, showing that patients treated with first line VEGF had an ORR of 29.6%, with no additional variables able to predict response in this subgroup. On the other hand, patients treated with IO-IO and IO-TKI had a combined ORR of 42.7%. The second variable to consider for this subgroup was whether they had a nephrectomy, showing an ORR of 35.0% for patients treated with IO-IO and IO-TKI with no nephrectomy and 46.9% for those with nephrectomy. Patients that underwent a nephrectomy were again divided by therapy type, showing an ORR of 42.9% for patients treated with IO-IO and prior nephrectomy and 54.9% for patients treated with IO-TKI that had a nephrectomy. The IO-IO and nephrectomy subgroup was further divided regarding the presence of lung metastasis, showing a poorer ORR (29.2%) for those without lung metastasis; and 47.1% for those with lung metastasis. The IO-IO, nephrectomy and lung metastasis subgroup was again divided regarding the presence of other metastasis, showing an ORR of 43.8% if there were other metastasis and 59.8% if there were only lung metastasis. Finally, the subgroup of patients with IO-TKI and nephrectomy was divided by age, showing an ORR of 43.6% for those older or equal than 70 years and 58.4% for those younger than 70 years.

To summarize, based on 5 hierarchical variables, CART analysis divided the population in 7 categories with different response probabilities.

1. VEGF.
2. IO-IO or IO-TKI and no nephrectomy.
3. IO-IO, nephrectomy and no lung metastasis.
4. IO-IO, nephrectomy, lung metastasis and other metastasis.
5. IO-IO, nephrectomy and only lung metastasis.
6. IO-TKI, nephrectomy and >70 years.
7. IO-TKI, nephrectomy and <70 years.

### Secondary endpoints

Secondary endpoints for each of these subgroups are summarized in **Table 3**. TD was longest in the nephrectomy IO-TKI groups (median 19.8-21.2 months), compared to 7.6 months in the VEGF group and 7.3 months in the IO-IO/IO-TKI non-nephrectomy group. TTNT varied widely, from 9.4 months (VEGF) to 39.2 months in the IO-IO, lung-only metastases group. Median OS was most favorable for the IO-TKI <70 age with nephrectomy group (91.1 months; 95% CI, 55.9–NA), followed by the IO-IO with no lung metastases group (54.5 months; 95% CI, 50.5–NA). The VEGF group had a median overall survival of 29.5 months (95% CI, 26.1–32.8). The 18-month survival probability was highest for the IO-IO subgroups with isolated lung metastases (92.8%, 95% CI, 86.9–99.1) and the IO-TKI with age <70 group (88.4%, 95% CI, 84.0–93.0). The overall cohort had a median survival of 35.4 months and an 18-month survival rate of 68.4%.

**Table 3:** Secondary outcomes.

Risk groups	N (%)	Odds ratio for ORR (95% CI)	Median TD in months (95% CI)	Median TTNT in months (95% CI)	Median survival in months (95% CI)	18 month survival rate (95% CI)
1) VEGF	1313 (51.5)	ref (1.0)	7.6 (7.0-8.2)	9.4 (8.6-10.3)	29.5 (26.1-32.8)	0.622 (0.595-0.650)
2) IO-IO or IO-TKI and no nephrectomy	443 (17.4)	1.28 (1.02-1.60)	7.3 (5.6-9.0)	10.2 (8.8-11.3)	22.4 (20.1-26.6)	0.597 (0.548-0.652)
IO-IO and nephrectomy						
3) No lung mets	137 (5.4)	0.98 (0.66-1.43)	10.1 (5.7-15.5)	17.2 (10.6-30.1)	54.5 (50.5-NA)	0.847 (0.783-0.916)
4) Lung met and met at other sites	267 (10.5)	1.87 (1.42-2.44)	7.2 (5.3-9.4)	13.0 (10.1-20.5)	47.1 (40.5-NA)	0.775 (0.723-0.830)
5) Only lung met	85 (3.3)	3.56 (2.28-5.63)	9.3 (6.2-16.8)	39.2 (14.4-NA)	50.5 (39.7-NA)	0.928 (0.869-0.991)
IO-TKI and nephrectomy						
6) Age 70+	78 (3.2)	1.84 (1.15-2.91)	21.2 (14.3-NA)	35.7 (19.8-NA)	44.5 (30.4-NA)	0.802 (0.710-0.907)
7) Age < 70	226 (8.9)	3.34 (2.50-4.47)	19.8 (16.4-23.5)	24.7 (22.4-36.4)	91.1 (55.9-NA)	0.884 (0.840-0.930)
Overall	2549		8.4 (7.9-9.1)	11.5 (10.7-12.2)	35.4 (32.9-39.1)	0.684 (0.665-0.704)

VEGF, Vascular endothelial growth factor receptor tyrosine kinase inhibitor; IO-IO, ipilimumab + nivolumab; IO-TKI, immunotherapy plus a vascular endothelial growth factor receptor tyrosine kinase inhibitor; met, metastasis.

## Discussion

This large, multicenter retrospective study used the IMDC and a ML based CART analysis to identify subgroups of patients with mRCC with differential ORR across first-line treatment regimens in the real-world setting. Its focus was not to build a predictive model for treatment selection. As such, the AUC of 0.58 observed in the test dataset reflects the exploratory and descriptive nature of this classification approach, and is not intended to supplant validated prognostic tools such as the IMDC risk score. This work represents an early application of machine learning methodology to this clinical question; further refinement and prospective validation will be required before clinical implementation. That being said, our findings highlight several important features that add nuance to the current treatment decision making paradigm in the increasingly complex world of kidney cancer.

First, as expected and consistent with results from pivotal randomized trials, immune based combinations, particularly IO-TKI regimens, were associated with the highest ORR (50.2%) and better long-term outcomes compared to VEGF monotherapy. While VEGF inhibitors retain activity, their response rates and survival outcomes were inferior, with no additional factor identified that predicted improved ORR, reflecting their historical role as a prior standard of care.

Second, this study emphasizes the continued relevance of clinical variables, most notably prior nephrectomy, lung-only metastasis, and younger age, in stratifying patients for expected benefit. The strong association between nephrectomy and higher response rates, aligns with prior analyses that shows poorer responses within the target renal tumor volume in pivotal trials (20), and other analyses, like one from the IMDC (21), showing better outcomes for patients with nephrectomies in the contemporary immunotherapy-based treatment era. Notably, patients treated with IO-IO who had isolated lung metastasis achieved exceptionally high response rates and prolonged time to next treatment and OS, supporting the hypothesis that this subgroup may derive long-term benefit from dual checkpoint inhibition. However, it’s worth mentioning that the IMDC risk was not a variable in this analysis, and that the IO-IO population is skewed towards the Intermediate and Poor risk because of real world approval of this combination, and ergo this should not be a taken as an endorsement of this approach in patients with favourable risk and lung only metastasis.

The IMDC individual risk score variables were in fact included in the initial CART modelling. However, it was retained only in more complex tree configurations and was not selected by the algorithm in the final simplified model, where other variables demonstrated superior discriminatory capacity. The decision to exclude the known IMDC risk variables from the final model was therefore data-driven, not by design, and is acknowledged as a limitation of the present analysis.

While the individual variables identified by the CART model are established prognostic factors in mRCC, the clinical value of this hierarchical analysis lies in elucidating the sequential and interactive relationships between these variables in determining ORR. Unlike additive risk scores such as IMDC or MSKCC, decision tree methodology captures non-linear interactions and differential weighting of variables across treatment subgroups, providing a complementary framework for understanding response heterogeneity in real-world practice.

Our findings also illustrate the capacity of ML tools to refine risk stratification beyond conventional IMDC scoring. While the IMDC model remains an important prognostic tool, CART analysis offers a pragmatic approach to uncovering complex interactions between treatment type, baseline characteristics, and outcomes. This approach can complement traditional multivariable models by identifying clinically intuitive subgroups that may inform shared decision-making.

Several caveats warrant mention. As a retrospective study, treatment allocation was not randomized, and physician or institutional preference may have influenced regimen selection, potentially introducing confounding. As an example, IO-IO use in favourable risk is low and can’t be generalized to that group based upon these results. Despite efforts to standardize data collection, variability in imaging assessments and response adjudication across centers is an inherent limitation. Moreover, CART-derived subgroups, while internally validated, require prospective confirmation before routine clinical implementation. Additionally, this CART analysis objective was to identify predictors of response, and response does not necessarily predict OS, which is the most important endpoint. Nevertheless, the size, global representation, and contemporary nature of this cohort strengthen the generalizability of our findings. Another significant limitation of this analysis is the inherent temporal and indication bias introduced by the inclusion of patients treated across a decade (2015–2024). Patients receiving VEGF-targeted monotherapy prior to the widespread adoption of IO-based combinations, are likely to represent a distinct patient population with different baseline risk profiles, comorbidities, and treatment indication patterns. Non-randomised treatment allocation in real-world datasets precludes direct comparison of outcomes across treatment groups, and the findings should be interpreted within this context.

Taken together, these results reinforce the value of tailoring therapy selection based not only on IMDC risk but also on readily available clinical features, such as prior nephrectomy and metastatic pattern, while highlighting the promise of ML in optimizing patient stratification in mRCC.

## Conclusion

CART-based modeling identified seven clinically meaningful subgroups with distinct probabilities of response, emphasizing the importance of integrating treatment selection with clinical features such as prior nephrectomy, metastatic pattern, and patient age. These findings underscore the utility of ML as a complement to established prognostic models, offering a framework for more individualized treatment strategies. Prospective validation of these subgroups is warranted to refine therapy selection and optimize outcomes for patients with mRCC.

## Data Availability

The data analyzed in this study is subject to the following licenses/restrictions: The data supporting this study originate from the International Metastatic Renal Cell Carcinoma Database Consortium (IMDC), a collaborative network with strict data governance policies. Due to confidentiality agreements, institutional review board requirements, and the terms of the consortium’s data sharing agreements, the dataset cannot be made publicly available. Qualified researchers may submit a formal request to the IMDC executive committee for data access, subject to institutional approvals and data use agreements. Requests to access these datasets should be directed to Daniel Heng, daniel.heng@albertahealthservices.ca.
